# Circulating miRNAs Correlate With rIPC‐Induced Cardioprotection and Its Impairment in Diabetic Myocardial Infarction via AMPK Signalling

**DOI:** 10.1111/jcmm.71163

**Published:** 2026-05-13

**Authors:** Yeyi Bai, Bang'e Zhao, Tingting Liu, Bei Tian, Haijian Liu

**Affiliations:** ^1^ Department of Anesthesiology Shanghai University of Medicine & Health Sciences Affiliated Zhoupu Hospital Shanghai China; ^2^ Department of Nursing Shanghai University of Medicine & Health Sciences Affiliated Zhoupu Hospital Shanghai China

**Keywords:** AMI, AMPK, miRNA, rIPC

## Abstract

Remote ischemic preconditioning (rIPC) has shown potential in protecting myocardial tissue against acute myocardial infarction (AMI), primarily through anti‐apoptotic, anti‐inflammatory, and anti‐hypoxic mechanisms. However, its effectiveness is compromised in the presence of metabolic comorbidities such as diabetes. Circulating microRNAs (miRNAs), which act as intercellular communicators, have been implicated in the systemic effects of rIPC, but their profiles and underlying mechanisms in diabetic conditions remain unclear. We constructed AMI models in both diabetic and non‐diabetic rats and performed rIPC treatment. Plasma samples were collected for miRNA sequencing to identify differentially expressed circulating miRNAs. We observed that rIPC significantly altered the expression of several circulating miRNAs, including miR‐19a‐3p, miR‐221‐5p and miR‐210‐5p, which were associated with improved cardiomyocyte survival under ischemic conditions. Conversely, miR‐34a‐3p, miR‐532‐5p and miR‐410‐5p were found to be up‐regulated in diabetic rats and were associated with enhanced cardiomyocyte apoptosis, inflammation and impaired rIPC efficacy. These miRNAs may be downstream of AMPK signalling, suggesting a potential molecular association between rIPC and metabolic status. This study identifies a panel of circulating miRNAs that are associated with the beneficial effects of rIPC and those that are linked to its impairment under diabetic conditions. Our findings highlight circulating miRNAs as candidate modulators that may influence myocardial responses under comorbid AMI. Nevertheless, the cellular source and upstream regulation of these miRNAs, as well as their effects on non‐cardiac organs, warrant further investigation.

## 1 Introduction

Acute myocardial infarction (AMI) is one of the most prevalent forms of ischemic heart disease and remains a leading cause of morbidity and mortality worldwide [1]. Despite advances in early diagnosis and reperfusion therapies, myocardial ischemia/reperfusion injury (IRI) continues to be a major limiting factor in the treatment of AMI [2]. IRI refers to a paradoxical exacerbation of tissue damage that occurs upon the restoration of blood flow following a period of ischemia. The sudden reperfusion induces a cascade of pathophysiological responses, including mitochondrial dysfunction, oxidative stress, calcium overload, inflammatory activation and apoptosis, which together aggravate myocardial injury [3]. Furthermore, reperfusion‐induced damage is not limited to the heart itself; it can also contribute to systemic inflammation and dysfunction of remote organs, potentially leading to multiple organ dysfunction syndrome (MODS) [4, 5]. To date, no clinically approved strategy has been able to effectively mitigate IRI or substantially improve outcomes in ischemic heart disease, highlighting the urgent need for novel cardioprotective approaches.

Previous studies have demonstrated that remote ischemic preconditioning (rIPC) can be effectively induced not only in limbs, but also in other organs and tissues such as the small intestine, kidneys and liver [6]. rIPC refers to the phenomenon whereby brief, non‐lethal cycles of ischemia and reperfusion applied to a remote organ or tissue—most commonly a limb—confer protection against subsequent IRI in distant vital organs including the heart, brain, liver and kidneys [7]. Although the underlying mechanisms are not yet fully elucidated, growing evidence suggests that rIPC exerts its protective effects through a multifaceted network involving neural pathways, humoral mediators and systemic immune modulation [8]. Activation of afferent sensory nerves during remote ischemia may initiate neurogenic signalling cascades that rapidly transmit protective signals to target organs. In parallel, rIPC stimulates the release of circulating mediators such as adenosine, bradykinin, nitric oxide and extracellular vesicles carrying microRNAs and cytoprotective proteins, which subsequently activate pro‐survival kinases and anti‐apoptotic signalling pathways in remote tissues [9]. Additionally, rIPC modulates systemic inflammatory responses by influencing immune cell activation and cytokine production, thereby enhancing the tissue's resilience to ischemic stress [10]. Collectively, these mechanisms form an intricate inter‐organ communication network that underpins the systemic protective effects of rIPC.

Although rIPC is a non‐invasive and relatively safe clinical strategy that has shown promising cardioprotective effects in experimental and early clinical studies, its efficacy appears to be limited or inconsistent in certain patient populations. Specifically, individuals with chronic comorbidities such as diabetes mellitus, chronic kidney disease, advanced age, peripheral vascular disease, or those in states of severe infection or postoperative stress may exhibit impaired responses to rIPC [11]. Emerging evidence suggests that several mechanisms may underlie this diminished efficacy. First, key pro‐survival signalling cascades such as the PI3K‐Akt [12] and STAT3 pathways [13], which are essential for transducing rIPC‐mediated cardioprotection, are often disrupted in chronic diseases—for example, insulin resistance in diabetes compromises PI3K‐Akt signalling [14, 15]. Second, endothelial dysfunction [16, 17] and decreased vascular compliance [18], commonly observed in hypertension and atherosclerosis, may hinder the effective transmission of protective signals from the remote site to the myocardium. Third, dysregulated humoral responses‐such as excessive release of pro‐inflammatory cytokines (e.g., IL‐6, TNF‐α) in settings of systemic inflammation or immune imbalance‐may exacerbate rather than ameliorate ischemia–reperfusion injury [19, 20, 21]. Lastly, impaired neural reflex arcs in aging or neuropathic patients may blunt the neurogenic signalling component of rIPC [22, 23]. Despite these challenges, ongoing research has proposed several strategies to enhance the effectiveness of rIPC. These include pharmacological agents that restore endothelial or mitochondrial function, optimization of rIPC protocols tailored to specific disease contexts, and the identification of reliable biomarkers to predict patient responsiveness [24, 25]. Therefore, addressing these limitations is essential to expand the clinical applicability of rIPC and to develop precision‐based cardioprotective strategies for high‐risk populations.

Accumulating evidence suggests that circulating microRNAs (miRNAs) play a critical role in mediating the systemic effects of rIPC, acting as key messengers in the communication between remote organs and the heart [26, 27, 28]. Several miRNAs have been identified to be up‐regulated or down‐regulated in response to rIPC, and some have been linked to anti‐apoptotic, anti‐inflammatory, or pro‐survival signalling in cardiomyocytes [7, 29]. However, the specific miRNA signatures associated with rIPC remain inconsistent across different pathological conditions, particularly in the presence of comorbidities such as diabetes [30, 31]. Moreover, the molecular mechanisms by which these miRNAs modulate cardiomyocyte survival in the context of rIPC remain largely unclear. Further research is needed to clarify the identity and functional relevance of these miRNAs and to determine how disease states may alter their regulatory roles.

In this study, we focused on circulating miRNAs to investigate the differential expression profiles mediated by rIPC in AMI rats with and without comorbid diabetes mellitus. We identified a subset of miRNAs that were either positively associated with the cardioprotective effects of rIPC or potentially responsible for attenuating its efficacy. Furthermore, we explored their potential molecular mechanisms in regulating cardiomyocyte apoptosis. These findings provide novel insights into the regulatory targets and signalling pathways involved in rIPC and offer promising strategies to optimize the clinical application of rIPC for the rescue of AMI patients, particularly those with underlying metabolic disorders.

## 2 Materials and Methods

### 2.1 Reagents

Detailed information of reagents used in this study was listed in Table S1.

### 2.2 Animal Experimental Design

A total of 60 adult male Sprague–Dawley (SD) rats (220–250 g) were randomly assigned to six groups: normal control (NC, *n* = 10), myocardial infarction (MI, *n* = 10), MI + rIPC (*n* = 10), diabetic MI (DMI, *n* = 10), DMI + rIPC (*n* = 10), and DMI + rIPC + AMPK agonist (AMPKA, *n* = 10). Randomization was performed prior to experimental procedures. Investigators responsible for outcome assessment were blinded to group allocation. Animals were excluded based on predefined criteria including: (1) unsuccessful induction of diabetes (fasting blood glucose < 16.7 mmol/L after STZ); (2) failure of AMI induction (absence of myocardial pallor and ST elevation); (3) perioperative death unrelated to experimental endpoints; (4) technical failure during sample processing. Excluded animals were replaced to maintain the predefined group size. In accordance with ARRIVE guidelines, the number of animals initially included, excluded, and surviving to the experimental endpoint in each group is summarized in Table S2. Sample size was determined based on previous studies using similar experimental models and endpoints, as well as preliminary data, to ensure sufficient statistical power to detect biologically relevant differences. No formal a priori power calculation was performed. All procedures were approved by the Ethics Committee of Shanghai Pudong New Area Zhoupu Hospital (ZPYYLL‐2018‐02) and conducted in accordance with relevant guidelines.

### 2.3 Disease and Intervention Model Construction Process

Diabetes was induced by a single intraperitoneal injection of 60 mg/kg streptozotocin (STZ) freshly prepared in 0.1 M citrate buffer [32]. Rats with fasting blood glucose levels ≥ 16.7 mmol/L after 72 h were considered diabetic.

Four weeks after diabetes induction, the AMI model was established by permanent ligation of the left anterior descending (LAD) coronary artery [33]. Briefly, rats were anaesthetised with an intraperitoneal injection of pentobarbital sodium (50 mg/kg), intubated, and ventilated with a rodent ventilator. Following a left thoracotomy at the fourth intercostal space, the heart was exteriorized, and the LAD was ligated approximately 2–3 mm from its origin using a 6–0 silk suture. Successful ligation was confirmed by regional myocardial pallor and ST‐segment elevation on electrocardiogram. The chest was then closed in layers, and the rats were allowed to recover under close monitoring. Sham‐operated rats underwent the same procedure without LAD ligation. rIPC was performed immediately following the induction of AMI.

rIPC was applied to the left hind limb of the rat using a rubber tourniquet tightly wrapped around the proximal thigh to occlude blood flow [34]. The occlusion was confirmed by observing pallor and reduced limb temperature distal to the tourniquet. The rIPC protocol consisted of three cycles of 5‐min ischaemia followed by 5‐min reperfusion (total duration: 30 min). During the reperfusion phase of each cycle, the tourniquet was released to restore blood flow to the limb. Throughout the procedure, the animals were maintained under anaesthesia, and body temperature was monitored and kept constant using a heating pad. In the DMI + rIPC + AMPKA group, 500 mg/kg AMPK agonist AICAR [35, 36, 37, 38] was administered intraperitoneally 30 min before the rIPC procedure.

### 2.4 Echocardiographic Assessment

Cardiac function was evaluated 24 h after AMI by transthoracic echocardiography [39] and invasive hemodynamic measurement [33]. For echocardiography, rats were lightly anaesthetised to maintain a stable heart rate and spontaneous breathing, placed in the supine position on a temperature‐controlled platform, and the chest was shaved. Two‐dimensional and M‐mode images were acquired from the parasternal short‐axis view at the level of the papillary muscles using a high‐frequency small‐animal ultrasound system. Left ventricular end‐diastolic diameter (LVEDD) and left ventricular end‐systolic diameter (LVESD) were measured from M‐mode tracings over at least three consecutive cardiac cycles by an investigator blinded to group allocation. Left ventricular fractional shortening (LVFS) was calculated as (LVEDD − LVESD)/LVEDD × 100, and left ventricular ejection fraction (LVEF) was automatically calculated by the ultrasound software or derived from standard formulas based on ventricular dimensions.

### 2.5 Hemodynamic Measurements

Immediately after echocardiography, invasive hemodynamic parameters were recorded. Deep anaesthesia was induced using pentobarbital sodium (150 mg/kg, intraperitoneal), a micro‐tip pressure catheter (e.g., 1.4F) was inserted into the left ventricle via the right carotid artery. After stabilization, left ventricular systolic pressure (LVSP) and left ventricular end‐diastolic pressure (LVEDP) were continuously recorded using a pressure transducer and data acquisition system. All measurements were performed under comparable anaesthetic depth and temperature control to minimize physiological variability.

### 2.6 Tissue Collection

All rats were sacrificed 24 h after the AMI procedure for sample collection. Prior to sacrifice, deep anaesthesia was induced by intraperitoneal injection of pentobarbital sodium (150 mg/kg), and the adequate anaesthetic depth was confirmed by the absence of pedal withdrawal reflex before tissue collection. All procedures were performed in accordance with institutional guidelines for the humane treatment of laboratory animals. Cardiac perfusion was performed to remove circulating blood. The hearts were then rapidly excised, and the anterior wall of the left ventricle was carefully dissected for further analyses. Part of the myocardial tissue was fixed in 4% paraformaldehyde for subsequent paraffin embedding and histological analysis. The remaining tissue was snap‐frozen in liquid nitrogen and stored at −80°C for RNA and protein extraction.

### 2.7 Enzyme‐Linked Immunosorbent Assay (ELISA)

Peripheral blood was collected from anaesthetised rats via cardiac puncture, and transferred into anticoagulant‐free tubes and allowed to clot at room temperature for 30 min. Serum was separated by centrifugation at 3000 rpm for 10 min, and stored at −80°C until analysis. ELISA was performed to quantify the levels of Tumour Necrosis Factor Alpha (TNF‐α), Interleukin 6 (IL‐6), Interleukin 1 Beta (IL‐1β), Creatine Kinase MB Isoenzyme (CK‐MB) and malondialdehyde (MDA) in the serum according to the manufacturer's instructions. Briefly, 100 μL of standards and appropriately diluted samples were added to the wells of a 96‐well microplate pre‐coated with a capture antibody and incubated at 37°C for 2 h. After washing with the provided wash buffer, 100 μL of biotin‐labelled detection antibody was added and incubated at 37°C for 1 h. The wells were then washed and incubated with 100 μL of HRP‐conjugated streptavidin for 30 min at 37°C. After a final washing step, 90 μL of TMB substrate solution was added to each well and incubated in the dark for 15–20 min. The reaction was stopped with 50 μL of stop solution, and absorbance was measured at 450 nm using a BioTek Synergey HTX microplate reader (Agilent Technologies, Santa Clara, CA, USA). All samples were assayed in duplicate.

### 2.8 Triphenyltetrazolium Chloride (TTC) Staining

To evaluate myocardial infarct size, 2,3,5‐triphenyltetrazolium chloride staining was performed. After euthanasia, the hearts were rapidly excised, rinsed in cold PBS, and frozen at −20°C for 10–15 min to facilitate slicing. The hearts were then transversely sectioned into 2‐mm thick slices from apex to base. The slices were incubated in 1% TTC solution prepared in PBS at 37°C for 20 min in the dark, with gentle agitation every 5 min to ensure uniform staining. Viable myocardium stained deep red, whereas infarcted tissue lacking dehydrogenase activity remained pale white. After staining, the slices were fixed in 4% paraformaldehyde for 30 min. Images of the slices were captured, and infarct size was quantified using ImageJ software by calculating the percentage of infarcted (white) area relative to the total myocardial area.

### 2.9 Haematoxylin and Eosin (H&E) Staining

The severity of myocardial infarction was assessed based on H&E staining. After the completion of experimental interventions, rat hearts were harvested, fixed in 4% paraformaldehyde for 48 h, and embedded in paraffin. Serial transverse sections (5 μm thick) were obtained from the apex to the base of the heart at 2–3 mm intervals. Sections were stained with H&E and visualized under a light microscope. The infarct size was evaluated based on morphological changes such as loss of myocardial striations, increased eosinophilic staining, nuclear pyknosis or disappearance, and inflammatory cell infiltration. Digital images of each section were captured, and the infarct area (non‐viable myocardium) and total left ventricular (LV) area were measured using ImageJ software. The infarct size was calculated as the percentage of infarct area relative to the total LV area for each section, and the average of all sections was used to represent the infarct size of each heart: $\text{Infarct size}\;\left(\%\right)=\frac{\text{Infarct area}}{\text{Total}\;\mathrm{LV}\;\text{area}}\times 100\%$.

### 2.10 Immunohistochemistry (IHC) Assay

Paraffin‐embedded heart tissue sections (5 μm thick) were deparaffinized in xylene and rehydrated through a graded ethanol series. Endogenous peroxidase activity was quenched by incubating the sections in 3% hydrogen peroxide for 10 min at room temperature. Antigen retrieval was carried out by heating the sections in citrate buffer (10 mM, pH 6.0) using a microwave or pressure cooker for 15 min. After cooling to room temperature, the sections were blocked with 5% normal goat serum for 30 min to reduce nonspecific binding. The samples were then incubated overnight at 4°C with the primary antibodies against HIF‐1α (1: 200), Bax (1: 1000), Bcl‐2 (1: 500) and IL‐10 (1: 500). After washing in PBS, sections were incubated with an HRP‐conjugated goat anti‐rabbit IgG (H + L) (1: 2000) for 30 min at room temperature. Colour development was achieved using diaminobenzidine (DAB) as the chromogen. The sections were counterstained with haematoxylin, dehydrated, cleared in xylene and mounted with neutral resin. Positive staining appeared brown and was evaluated under a light microscope.

### 2.11 RNA Isolation

Total RNA was extracted from freshly isolated myocardial tissue using TRIzol reagent according to the manufacturer's instructions. RNA quantity and purity were assessed using a NanoDrop One/One spectrophotometer (Thermo Fisher Scientific), and integrity was evaluated by agarose gel electrophoresis and the Agilent 2100 Bioanalyzer (Agilent Technologies).

### 2.12 Quantitative Real‐Time PCR (qPCR)

Reverse transcription was carried out using a miRNA‐specific miScript II RT Kit system. qPCR amplification was performed using miScript SYBR Green PCR Kit on a QuantStudio real‐time PCR system. Primers were purchased from miScript Primer of QIAGEN. Each reaction was performed in triplicate, and no‐template controls were included to exclude contamination. Relative miRNA expression levels were calculated using the 2^−ΔΔCt^ method. For normalization, U6 small nuclear RNA was used as an endogenous internal control. In addition, a synthetic spike‐in control (cel‐miR‐39) was added during RNA extraction to control for technical variation in extraction and reverse transcription efficiency. Normalization of miRNA expression is essential to reduce technical variation and ensure accurate quantification. The use of reference genes of U6 is widely applied in miRNA qPCR studies to correct for experimental variability and enable reliable comparison between samples.

### 2.13 miRNA Sequencing

Only samples with RNA Integrity Number (RIN) ≥ 7.0 were used for subsequent library construction. Reverse transcription was performed using random hexamer primers and reverse transcriptase to synthesize cDNA using Ion Torrent NGS Reverse Transcription Kit. Three independent biological replicates were included for RNA sequencing. Libraries were prepared using Collibr Stranded RNA Library Prep Kit for Illumina Systems with Human/Mouse/Rat rRNA Depletion Kit following the manufacturer's protocol. The libraries were quantified, pooled, and sequenced on a NovaSeq 6000 platform to generate paired‐end reads of 150 bp. An average sequencing depth of approximately 20 million reads per sample was achieved. Raw sequencing data were processed using fastp for quality control, and high‐quality reads were aligned to the reference genome of *Rattus norvegicus* Rnor 6.0 using HISAT2. Transcript abundance was quantified with featureCounts, and differential gene expression analysis was performed using DESeq2. Genes with an adjusted *p*‐value < 0.05 and |log_2_ fold change| > 1 were considered differentially expressed.

For miRNA sequencing [40], circulating miRNAs were extracted from rat serum samples using the *miRNeasy Serum/Plasma Kit* according to the manufacturer's instructions. Briefly, 200 μL of serum was lysed and mixed with QIAzol reagent, followed by phase separation with chloroform and RNA precipitation using ethanol. The RNA was then purified on a spin column, eluted in RNase‐free water, and quantified using the Qubit microRNA Assay Kit. RNA integrity and small RNA size distribution were assessed with the Agilent 2100 Bioanalyzer. Three independent biological replicates were included for miRNA sequencing. Small RNA libraries were prepared using the NEBNext Small RNA Library Prep Set for Illumina. Briefly, 3′ and 5′ adapters were ligated to the small RNAs, followed by reverse transcription and PCR amplification with barcoded primers. Size selection of 140–160 bp cDNA fragments (corresponding to 20–40 nt RNA inserts) was carried out using polyacrylamide gel electrophoresis. The purified libraries were quantified, pooled in equimolar amounts, and sequenced on the Illumina NovaSeq 6000 platform with single‐end 50 bp reads. An average sequencing depth of approximately 10 million reads per sample was achieved. Raw sequencing data were processed to remove adaptors and low‐quality reads, and clean reads were mapped to the *miRBase* database for miRNA identification and expression analysis. MiRNA expression levels were normalized using the counts per million method to account for differences in sequencing depth across samples. Differential expression analysis was performed using DESeq2, and statistical significance was determined after Benjamini‐Hochberg false discovery rate (FDR) correction for multiple testing. MiRNAs with an adjusted *p* value < 0.05 were considered significantly differentially expressed. To minimize potential batch effects, all samples were processed using the same library preparation protocol and sequenced on the same platform. In addition, principal component analysis was performed to assess sample clustering and confirm the absence of significant batch‐related variability.

### 2.14 Cell Culture

H9c2 rat cardiomyoblast cells were cultured in high‐glucose Dulbecco's Modified Eagle Medium (DMEM) supplemented with 10% fetal bovine serum (FBS) and 1% penicillin–streptomycin at 37°C in a humidified atmosphere containing 5% CO_2_. For hypoxia treatment, cells were transferred to a Heracel VIOS 160i incubator (Thermo Fisher Scientific) with 1% O_2_, 5% CO_2_ and 94% N_2_ for 24 h as specified in each experiment.

For miRNA overexpression or disruption, cells were transfected with miRNA mimics at a final concentration of 50 nM using Lipofectamine 3000 according to the manufacturer's protocol. The miRNA sponge sequences were designed to contain multiple tandem binding sites complementary to the target miRNA, synthesized and cloned into the pCDH‐CMV‐MCS‐EF1‐puro lentiviral vector. Lentivirus was produced in HEK293T cells, and H9c2 cells were infected with lentiviruses at a multiplicity of infection (MOI) of 10 in the presence of 8 μg/mL Polybrene. After 24 h, the medium was replaced with fresh complete medium. Lentiviral transduction efficiency was evaluated based on the percentage of fluorescent marker‐positive cells (Figure S1). Stable sponge‐expressing cells were achieved 72 h post‐infection, followed by 2 μg/mL puromycin for 5 days.

### 2.15 Terminal Deoxynucleotidyl Transferase dUTP Nick End Labelling (TUNEL) Staining

1 × 10^6^ H9c2 cells were seeded onto coverslips and treated as indicated. After treatment, the cells were fixed with 4% paraformaldehyde for 30 min at room temperature, followed by permeabilization with 0.1% Triton X‐100 in PBS for 5 min on ice. Apoptotic cells were detected using a Click‐iT Plus TUNEL assay kit according to the manufacturer's instructions. Briefly, cells were incubated with TUNEL reaction mixture for 1 h at 37°C in a humidified dark chamber. After washing with PBS, nuclei were counterstained with DAPI. Fluorescent images were captured using a fluorescence microscope and TUNEL‐positive cells were quantified using ImageJ software.

### 2.16 Western Blot

Total protein was extracted from 1 × 10^6^ H9c2 cells using RIPA lysis buffer containing protease and phosphatase inhibitors. Protein concentrations were determined using Pierce Dilution‐Free Rapid Gold BCA Protein Assay Kit. Equal amounts of protein (40 μg per lane) were separated by SDS‐PAGE and transferred onto PVDF membranes. After blocking with 5% non‐fat milk in TBST for 1 h at room temperature, membranes were incubated overnight at 4°C with primary antibodies against Bcl‐2 (1: 2000), IL‐10 (1: 2000), HIF‐1α (1: 2000), Bax (1: 2000), p‐AMPKα (Thr172) (1: 1000), AMPKα1 (1: 2000), LC3 (1: 2000), p‐mTOR (Ser2448) (1: 2000), mTOR (1: 2000), p62 (1: 2000) and GAPDH (1: 5000). After washing, membranes were incubated with HRP‐conjugated secondary antibodies for 1 h at room temperature. Immunoreactive bands were visualized using Ultra High Sensitivity ECL Kit and imaged with a Tanon Chemi Dog Automatic Luminescence Imaging System (Cat. No. 5200T, Tanon, Shanghai, China). Band intensities were quantified using ImageJ software, and normalized to GAPDH.

### 2.17 Immunofluorescence Staining

1 × 10^6^ H9c2 cells were fixed with 4% paraformaldehyde for 15 min at room temperature, followed by permeabilization with 0.1% Triton X‐100 for 10–20 min. After blocking with 5% bovine serum albumin (BSA) or normal goat serum for 1 h at room temperature, the sections were incubated overnight at 4°C with primary antibodies against Bcl‐2 (1:1000) and Bax (1:1000). After washing with PBS, the samples were incubated with CoraLite Plus 488‐Goat anti‐rabbit recombinant secondary antibodies (1:5000) and CoraLite Plus 594‐Goat anti‐mouse recombinant secondary antibodies (1:5000) for 1 h at room temperature in the dark. Cell nuclei were counterstained with DAPI for 10 min. Images were captured using a fluorescence microscope.

### 2.18 Statistical Analysis

All experimental data are presented as mean ± standard deviation (SD). Biological replicates refer to independent animals or independently treated cell cultures, whereas technical replicates refer to repeated measurements of the same sample. Normality of the data was assessed using the Shapiro–Wilk test. For comparisons between two groups, unpaired two‐tailed Student's *t*‐test was used for normally distributed data, and the Mann–Whitney *U* test was applied for non‐normally distributed data. For multiple group comparisons, one‐way analysis of variance (ANOVA) was performed for normally distributed data, followed by Tukey's post hoc test, while the Kruskal–Wallis test with Dunn's post hoc correction was used for non‐normally distributed data. Multiple testing correction was applied where appropriate. All statistical analyses were conducted using GraphPad Prism 9.0 (GraphPad Software, San Diego, CA, USA). A *p*‐value < 0.05 was considered statistically significant.

## 3 Results

### 3.1 The Cardioprotective Anti‐Apoptotic Effect of rIPC Is Attenuated in Diabetic Rats With Myocardial Infarction

**FIGURE 1:**
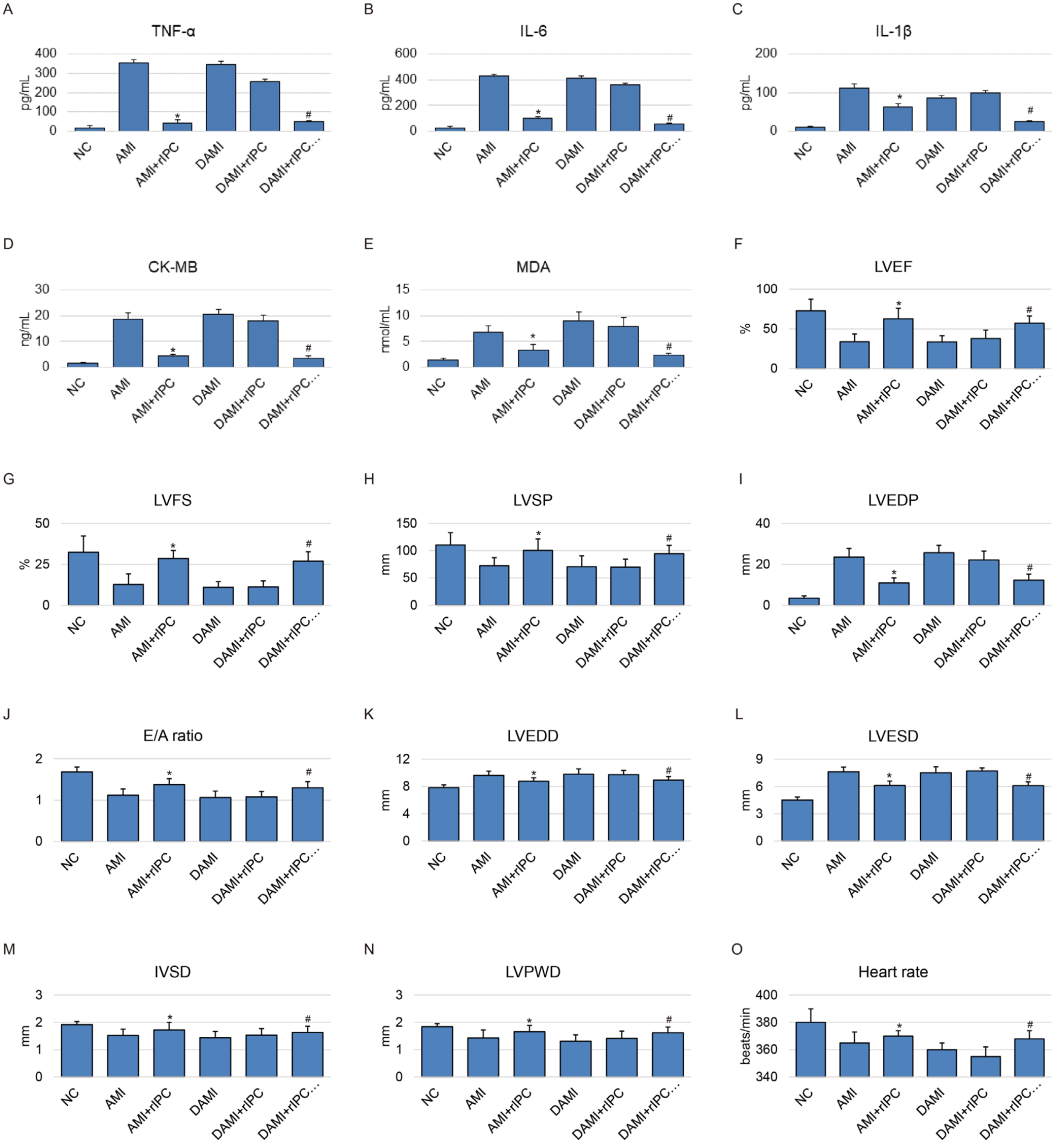
Remote ischemic preconditioning (rIPC) attenuates myocardial injury after acute myocardial infarction (AMI) in normal rats but not in diabetic rats. Rats were divided into six groups—NC, AMI, AMI + rIPC, AMI + diabetes (DAMI), AMI + diabetes + rIPC, and AMI + diabetes + rIPC + AMPK agonist (AMPKA). AMI was induced, and rIPC was performed via hind limb ischemia. (A‐E) Plasma levels of inflammatory and injury markers including TNF‐α (A), IL‐6 (B), IL‐1β (C), CK‐MB (D) and MDA (E) as well as (F–O) cardiac function parameters, including left ventricular ejection fraction (LVEF) and fractional shortening (LVFS), accompanied by left ventricular systolic pressure (LVSP) and left ventricular end‐diastolic pressure (LVEDP), as well as diastolic function early‐to‐late diastolic transmitral flow velocity ratio (E/A ratio), cardiac dimensions left ventricular end‐diastolic diameter (LVEDD) and left ventricular end‐systolic diameter (LVESD), wall thickness interventricular septal thickness at diastole (IVSD) and left ventricular posterior wall thickness at diastole (LVPWD), and heart rate (beats/min) were measured post‐AMI. In normal AMI rats, rIPC significantly reduced these markers, whereas in diabetic AMI rats, rIPC failed to elicit protective effects. Notably, AMPK agonist administration restored the cardioprotective efficacy of rIPC in diabetic rats, as evidenced by decreased levels of all measured markers. Data are presented as mean ± SEM (*n* = 10 per group, biological replicates). Each measurement was performed in triplicate. *p < 0.05 versus AMI group (normal); #*p* < 0.05 versus DAMI + rIPC.

To evaluate the cardioprotective effects of rIPC, we performed AMI surgery on both normal and diabetic rats, followed by rIPC intervention on the hind limb. In normal AMI rats, rIPC significantly attenuated myocardial injury, as evidenced by decreased plasma levels of inflammatory markers including TNF‐α (Figure 1A), IL‐6 (Figure 1B), IL‐1β (Figure 1C), CK‐MB (Figure 1D) and MDA (Figure 1E) compared to the AMI group. Consistently, echocardiographic and haemodynamic assessments demonstrated a marked improvement in cardiac function, with significantly increased left ventricular ejection fraction (LVEF) and fractional shortening (LVFS), accompanied by elevated left ventricular systolic pressure (LVSP) and reduced left ventricular end‐diastolic pressure (LVEDP) in the rIPC‐treated AMI rats (Figure 1F–I). In contrast, in diabetic AMI rats, rIPC failed to exert significant protective effects. No significant differences were observed in plasma TNF‐α, IL‐6, IL‐1β, MDA, or CK‐MB levels between diabetic AMI and diabetic AMI + rIPC groups. Similarly, rIPC did not significantly improve cardiac functional parameters in diabetic rats, as reflected by comparable LVEF, LVFS, LVSP, LVEDP, E/A ratio, LVEDD, LVESD, IVSD, LVPWD and heart rate values between these two groups (Figure 1F–O).

**FIGURE 2:**
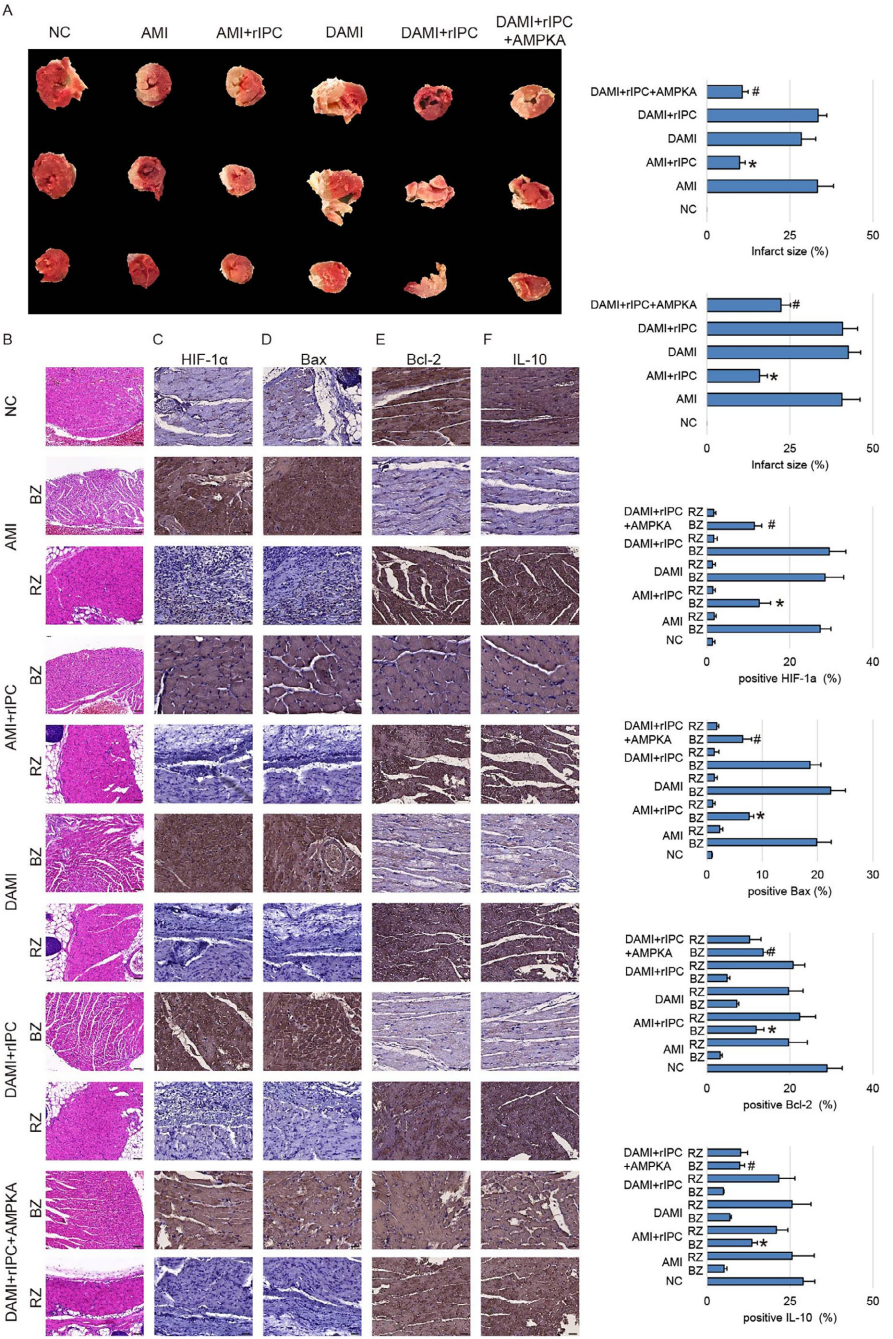
rIPC reduces myocardial infarction area and regulates hypoxia‐, apoptosis‐, and inflammation‐related markers in normal but not diabetic rats. (A, B) Representative TTC (A) and H&E staining (B) of border zone (BZ) and remote zone (RZ) of myocardial tissue from six groups: NC, AMI, AMI + rIPC, AMI + diabetes (DAMI), AMI + diabetes + rIPC and AMI + diabetes + rIPC + AMPK agonist (AMPKA) (*n* = 10 in each group). rIPC significantly reduced infarct area in normal rats with AMI but failed to do so in diabetic rats. However, administration of an AMPK agonist restored the infarct‐reducing effect of rIPC in diabetic rats. Scale bar, 50 μm. (C–F) Immunohistochemical staining for HIF‐1α (C), Bax (D), Bcl‐2 (E) and IL‐10 (F) in myocardial tissues. Scale bar, 50 μm. In normal rats, rIPC decreased the expression of HIF‐1α and Bax and increased the expression of Bcl‐2 and IL‐10, indicating attenuation of hypoxia, apoptosis and inflammation. However, in diabetic rats, these expression changes were not significant between the rIPC and AMI groups. Data are presented as mean ± SEM (*n* = 10 per group, biological replicates). Each experiment includes three technical replicates. *p < 0.05 versus AMI group (normal); #*p* < 0.05 versus DAMI + rIPC. BZ, border zone; RZ, remote zone.

Furthermore, the severity of myocardial infarction was also assessed based on TTC, H&E staining and IHC. Infarcted myocardium exhibited a characteristic pale appearance, indicating irreversible tissue necrosis, whereas viable myocardium was stained red. Notably, the border zone was identified as a transitional region between the infarct core and the remote myocardium, displaying heterogeneous TTC staining (Figure 2A). This region retained partial viability, consistent with metabolically compromised but structurally preserved cardiomyocytes, a hallmark of active cardiac remodelling. In contrast, the remote region exhibited uniform red staining, indicative of preserved myocardial viability and relatively intact tissue architecture. H&E staining revealed that in normal rats, the infarct area of myocardial tissue was significantly reduced in the rIPC‐treated group compared to the AMI group, indicating a protective effect of rIPC (Figure 2B). However, in diabetic rats, rIPC failed to reduce infarct size, with no significant difference observed between the rIPC and AMI groups. Likewise, immunohistochemical analysis showed that the expression levels of HIF‐1α (Figure 2C) and Bax (Figure 2D) were attenuated whereas the expression levels of Bcl‐2 (Figure 2E) and IL‐10 (Figure 2F) were elevated in the border zones of rIPC group relative to the AMI group in normal rats, suggesting that rIPC could mitigate hypoxia, inflammation, and apoptosis of cardiomyocytes. In contrast, these regulatory effects were not observed in diabetic rats, as rIPC treatment did not significantly alter the expression of these markers in the border zones compared to untreated AMI controls, indicating an impaired cardioprotective response under diabetic conditions. On the other hand, the expression of HIF‐1α, IL‐10, Bax and Bcl‐2 showed no significant difference in remote zone among these groups. Taken together, we established a diabetic rat model and subjected it to AMI and rIPC intervention to validate a clinically observed phenomenon: while rIPC exerts a cardioprotective effect against AMI‐induced injury, this protective effect is markedly diminished under underlying pathological conditions such as diabetes.

### 3.2 Characterization of Serum Circulating miRNA Profiles in Diabetic Rats With AMI Intervened by rIPC

**FIGURE 3:**
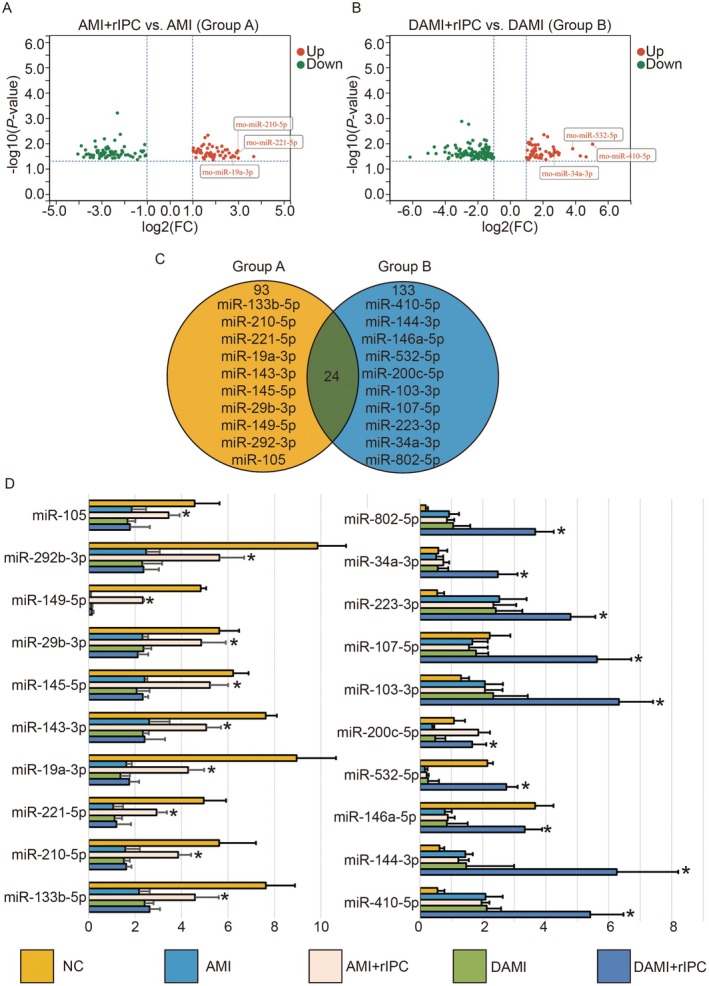
Identification of differentially expressed circulating miRNAs involved in the cardioprotective effect of rIPC under normal and diabetic conditions. (A, B) Volcano plots showing differentially expressed miRNAs between AMI + rIPC vs. AMI (Group A) and diabetic AMI + rIPC vs. diabetic AMI (Group B), respectively. Red dots indicate significantly up‐regulated miRNAs, and blue dots indicate down‐regulated miRNAs (*p* < 0.05, |log_2_FC| > 1). (C) Venn diagram illustrating the overlap and distinct sets of miRNAs between Group A and Group B comparisons. A total of 93 miRNAs were uniquely regulated in Group A and considered potential mediators of rIPC‐induced cardioprotection, while 133 miRNAs were uniquely regulated in Group B, possibly contributing to the diminished rIPC effects in diabetic rats. (D) Bar graph showing qPCR validation results of 20 miRNAs (top 10 with highest fold change from each group). qPCR data were consistent with sequencing results, confirming the reliability of miRNA expression profiles. Data are presented as mean ± SEM (*n* = 3 per group, biological replicates). Each experiment includes three technical replicates. *p < 0.05.

To further investigate the molecular mechanisms underlying the systemic cardioprotective effect of limb rIPC, we collected peripheral blood samples from four groups of rats (AMI, AMI + rIPC, diabetic AMI and diabetic AMI + rIPC) and performed miRNA sequencing in search of potential critical regulatory biomarkers. 118 differentially expressed miRNAs in the AMI + rIPC vs. AMI comparison (Group A) and 162 in the diabetic AMI + rIPC vs. diabetic AMI comparison (Group B) were identified (*p* < 0.05, |log_2_FC| > 1, Figure 3A,B, Tables S3, S4). Intersection analysis between the two sets revealed 93 miRNAs uniquely regulated in Group A, which were considered as potential mediators of rIPC‐induced cardioprotection. In contrast, 133 miRNAs were uniquely present in Group B, suggesting their possible involvement in the attenuation of rIPC benefits under diabetic conditions (Figure 3C). Among these group‐specific miRNAs, a total of 20 miRNAs ranked in the top 10 respectively in group A and Group B with the highest fold change were selected for qPCR validation. The qPCR results were consistent with the sequencing data (Figure 3D), further supporting the reliability of the differential expression profiles and their potential relevance to the cardioprotective effects of rIPC.

### 3.3 Identification of Circulating miRNA With Significant Impact on Cardioprotection In Vitro

**FIGURE 4:**
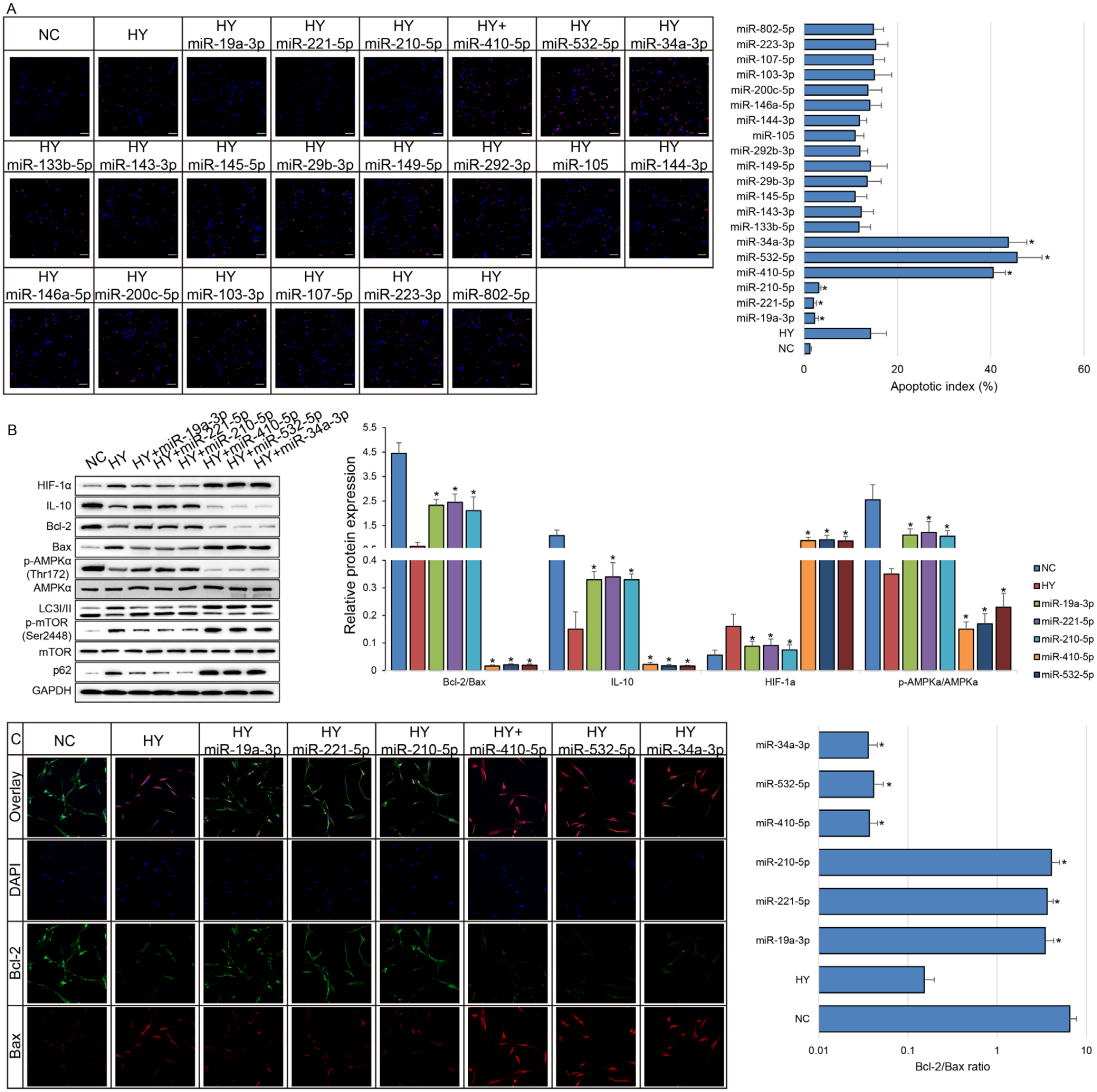
Functional validation of candidate miRNAs in regulating hypoxia‐induced apoptosis in cardiomyocytes. (A) TUNEL staining of H9c2 cardiomyocytes transfected with individual miRNA mimics and subjected to hypoxic conditions. Overexpression of Group A miRNAs (miR‐19a‐3p, miR‐221‐5p and miR‐210‐5p) significantly reduced hypoxia‐induced apoptosis, while Group B miRNAs (miR‐410‐5p, miR‐532‐5p and miR‐34a‐3p) markedly increased apoptosis. Scale bar: 50 μm. (B) Western blot analysis of HIF‐1α, IL‐10, Bcl‐2, Bax, AMPKα, LC3, mTOR and p62 expression as well as phospho‐AMPKα (Thr172) and phosphor‐mTOR (Ser2448) levels in H9c2 cells following transfection and hypoxic treatment. Quantification of protein expression levels normalized to GAPDH. (C) IF assay of Bcl‐2 and Bax in H9c2 cells following transfection and hypoxic treatment. Scale bar: 20 μm. The expression patterns or ratio were consistent with the respective anti‐apoptotic or pro‐apoptotic roles of the miRNAs. Data are presented as mean ± SEM (*n* = 3 per group, biological replicates). Each experiment includes three technical replicates. *p < 0.05 vs. control group.

To further validate the functional relevance of the 20 candidate miRNAs, miRNA mimics were administered individually in the rat cardiomyocyte cell line H9c2, followed by hypoxic culture to simulate ischemic conditions. TUNEL staining revealed that three miRNAs miR‐19a‐3p, miR‐221‐5p and miR‐210‐5p from Group A significantly inhibited hypoxia‐induced apoptosis, whereas three miRNAs miR‐410‐5p, miR‐532‐5p and miR‐34a‐3p from Group B markedly promoted apoptosis (Figure 4A). Consistent with these findings, the expression levels of HIF‐1α and IL‐10 (Figure 4B) as well as the Bax/Bcl‐2 ratio (Figure 4B,C) were altered in patterns that corroborated the anti‐hypoxic, anti‐apoptotic and anti‐inflammatory effects of the corresponding miRNAs. Moreover, AMPK downstream readouts, including the expression of p62, phosphorylation of mTOR and LC3‐II/I ratio, all determined that AMPK was functionally activated by miR‐19a‐3p, miR‐221‐5p and miR‐210‐5p (Figure 4B). These results suggested that specific circulating miRNAs induced by rIPC might modulate cardiomyocyte survival under hypoxic stress, and another subset of diabetic‐induced circulating miRNA may disturb the cardioprotective effect of rIPC.

### 3.4 Identification of Crucial Signalling Pathways in Cardiomyocytes Affected by rIPC

**FIGURE 5:**
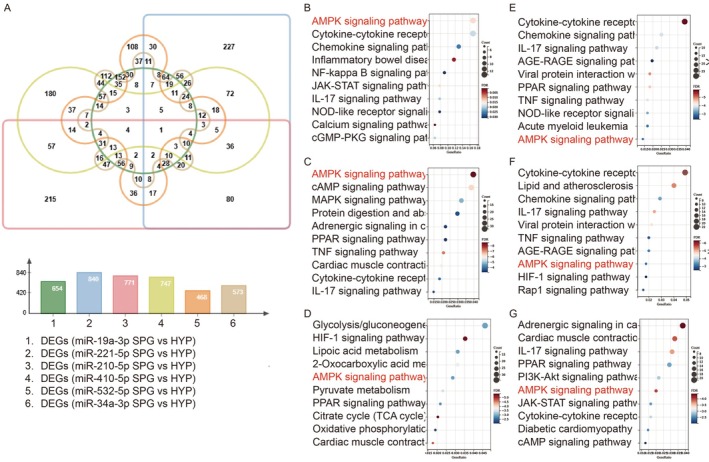
Transcriptomic and molecular analysis of miRNA function in H9c2 cardiomyocytes. (A) Venn diagram showing the overlap of differentially expressed genes (DEGs) following transfection of miRNA sponges targeting six candidate miRNAs in H9c2 cells, as identified by RNA sequencing. The limited overlap suggests distinct target profiles for each miRNA. (B–G) KEGG pathway enrichment analysis of each set of DEGs induced by miR‐19a‐3p (B), miR‐221‐5p (C), miR‐210‐5p (D), miR‐410‐5p (E), miR‐532‐5p (F), miR‐34a‐3p (G) sponges, revealing predominant enrichment in the AMPK signalling pathway.

Moreover, specific miRNA sponges targeting six candidate miRNAs were respectively transfected into H9c2 cardiomyocytes with hypoxic condition, followed by RNA sequencing. Differentially expressed genes (DEGs) induced by miRNA sponges were generated compared with hypoxic H9c2 cells (*p* < 0.05, |log_2_FC| > 1, Tables S5–, S10). Although the number of overlapping DEGs was limited (Figure 5A), suggesting that these miRNAs regulated distinct target genes, KEGG analysis revealed that each set of DEGs was all predominantly associated with the AMPK signalling pathway (Figure 5B–G). Additionally, DEGs were also involved in inflammatory response, cytokines receptor interaction, chemokine as well as other signalling pathways including JAK–STAT, MAPK, IL‐17, NF‐κB, HIF‐1, Rap1, cAMP. Western blot analysis further confirmed that miR‐19a‐3p, miR‐221‐5p and miR‐210‐5p suppressed, while miR‐410‐5p, miR‐532‐5p, miR‐34a‐3p enhanced the Thr172 phosphorylation of the AMPKα (Figure 4B). These findings suggested that both rIPC and diabetes might ultimately modulate cardioprotection through the regulation of this pathway. Notably, administration of an AMPK agonist in diabetic rats strengthened the protective effects of rIPC, supporting its pivotal role in this context (Figures 1 and 2).

Overall, our data suggested two sets of miRNAs contributing to rIPS‐mediated cardioprotection and diabetic‐induced anti‐rIPS effect.

## 4 Discussion

AMI remains one of the leading causes of morbidity and mortality worldwide, primarily due to irreversible myocardial damage caused by prolonged ischemia. Ischemic preconditioning (IPC), a phenomenon first described in the 1980s, refers to the process by which brief episodes of ischemia and reperfusion render the myocardium more resistant to a subsequent prolonged ischemic insult. Building on this concept, rIPC was later introduced as a less invasive strategy, in which transient ischemia is applied to distant organs or limbs to confer cardioprotection. Both IPC and rIPC have been shown to activate endogenous protective signalling pathways, including the PI3K/Akt and AMPK cascades, and are associated with reduced myocardial apoptosis, inflammation, and oxidative stress. Although the cardioprotective effects of rIPC have been consistently demonstrated in preclinical studies, clinical translation remains challenging. Variability in patient populations, comorbidities (such as diabetes and hypertension), and concurrent medications may interfere with the efficacy of rIPC in clinical settings. Nevertheless, ongoing trials and mechanistic studies continue to refine the application of IPC and rIPC as potential adjunctive therapies to improve outcomes in AMI patients undergoing reperfusion therapies.

Despite the promising cardioprotective effects of remote rIPC observed in animal models and healthy individuals, its clinical efficacy in AMI patients with comorbidities such as diabetes, hypertension, hyperlipidemia and aging remains limited. Accumulating evidence suggests that these underlying conditions impair the molecular signalling pathways activated by rIPC, thereby diminishing its protective potential. Among the key protective cascades triggered by rIPC, the PI3K/Akt pathway plays a pivotal role in promoting cell survival, inhibiting apoptosis and maintaining mitochondrial function. Activation of PI3K leads to phosphorylation of Akt, which subsequently enhances the expression of anti‐apoptotic proteins such as Bcl‐2 and inhibits pro‐apoptotic factors like Bax and Bad. However, hyperglycemia and insulin resistance in diabetic patients have been shown to attenuate PI3K/Akt signalling, reducing its cardioprotective efficacy [41]. Similarly, the MAPK/ERK pathway contributes to cellular survival and proliferation in response to ischemic stimuli [42]. ERK1/2 activation modulates mitochondrial permeability transition pore (mPTP) opening [43] and regulates genes such as HSP70 and eNOS [44], which are associated with cytoprotection. In hypertensive or aged myocardium, chronic oxidative stress and endothelial dysfunction can blunt ERK phosphorylation, limiting the benefits of rIPC. The JAK/STAT signalling pathway is another crucial mediator, especially STAT3, which is rapidly activated during rIPC and orchestrates anti‐inflammatory and anti‐apoptotic gene expression, including SOCS3, Bcl‐xL and MnSOD [45, 46, 47]. Inflammatory states or elevated cytokine environments, as seen in metabolic syndrome or chronic kidney disease, may desensitize or dysregulate JAK/STAT signalling, thereby weakening rIPC's effect. The cGMP/PKG pathway, which is downstream of nitric oxide (NO) production, regulates vascular tone and mitochondrial function [48]. Activation of soluble guanylyl cyclase (sGC) leads to increased cGMP levels, which activate protein kinase G (PKG) to inhibit mPTP opening and promote cardiomyocyte survival. However, oxidative stress and endothelial dysfunction in diabetic or obese patients reduce NO bioavailability and sGC responsiveness, impairing cGMP/PKG signalling and limiting rIPC‐induced protection. Furthermore, the TLR4/NF‐κB inflammatory pathway, typically suppressed by rIPC, is often up‐regulated in comorbid conditions [49]. rIPC is known to attenuate TLR4 expression and subsequent NF‐κB activation, thereby reducing pro‐inflammatory cytokines like TNF‐α and IL‐6. However, in patients with systemic inflammation, TLR4 may remain constitutively active, overriding rIPC's suppressive effects and promoting myocardial injury [50]. To overcome these limitations, recent studies have proposed several adjunctive strategies. These include pharmacological post‐conditioning with agents such as statins, GLP‐1 receptor agonists and PDE‐5 inhibitors to enhance key cardioprotective pathways. Gene therapy approaches targeting PI3K/Akt or STAT3 have also shown promise in preclinical models. Moreover, combining rIPC with exercise, caloric restriction, or antioxidant therapy may help restore pathway responsiveness and improve outcomes in patients with comorbid AMI.

AMPK is a key cellular energy sensor that maintains energy homeostasis by responding to changes in the intracellular AMP/ATP ratio. Upon activation, typically under metabolic stress or ischemic conditions, AMPK promotes catabolic pathways to generate ATP while inhibiting anabolic processes that consume energy. Functionally, AMPK enhances glucose uptake, fatty acid oxidation and mitochondrial biogenesis, while suppressing lipid synthesis, protein synthesis via mTOR inhibition and cell proliferation. Through these actions, AMPK plays a critical role in cardiovascular protection, metabolic regulation and cellular stress adaptation, making it a central signalling node associated with ischemia‐related pathophysiological processes [51, 52].

In the brain, rIPC significantly increased phosphorylated AMPK, along with HIF‐1α and HSP70; blockers of AMPK abolished rIPC‐mediated neuroprotection, indicating the involvement of the HIF‐1α/AMPK/HSP70 axis [53]. In a rodent focal ischemia model, rIPC elevated p‐AMPK levels 2.5‐fold and enhanced eNOS phosphorylation, while reducing infarct size and apoptosis, supporting a joint AMPK/eNOS mechanism in rIPC neuroprotection [54]. For cardiac tissue, acute rIPC stimuli trigger a rapid rise in phosphorylated AMPK, which suppresses mTOR activity and promotes autophagy (e.g., increased LC3‐II/I), suggesting AMPK activation is associated with autophagy‐related processes during early cardioprotection by rIPC [55]. Moreover, although IPC in hearts can occur independently of AMPK activation, genetic AMPK deficiency abolishes pharmacological preconditioning, suggesting that AMPK activation may be associated with certain aspects of IPC‐associated cardioprotection [56]. In diabetic hearts, AMPK activation in response to ischemia or ischemic post‐conditioning (IPO) is significantly attenuated, correlating with reduced autophagy, impaired mPTP inhibition, and loss of preconditioning benefits [57]. Importantly, pharmacological activation of AMPK is associated with improved IPO efficacy in diabetic models by re‐inducing autophagy, reducing infarct size and improving mitochondrial stability [58]. These findings suggest that AMPK is closely associated with rIPC‐induced tissue protection, potentially involving autophagy regulation, eNOS activation and mPTP inhibition. However, in the presence of metabolic diseases‐especially diabetes, which impairs AMPK activation‐rIPC efficacy is substantially reduced. These observations suggest that combinatorial strategies such as pharmacologic AMPK activation may help improve pathway responsiveness and enhance rIPC efficacy.

In recent years, circulating miRNAs have emerged as important components and potential biomarkers in cardiovascular diseases, offering new insights into intercellular communication under stress conditions such as AMI, ischemia–reperfusion injury and diabetes [59]. In this study, we identified a subset of blood‐borne miRNAs (miR‐19a, miR‐221 and miR‐210) that are up‐regulated following rIPC and are associated with its cardioprotective effects. Conversely, we found that miR‐410‐5p, miR‐532 and miR‐34a are up‐regulated in the diabetic state and likely attenuate the protective effect of rIPC on myocardial tissue, highlighting a possible molecular interaction between diabetes and cardioprotection failure. MiR‐19a has been previously associated with cardiomyocyte survival through inhibition of PTEN and activation of the PI3K/Akt pathway, thereby reducing apoptosis and oxidative stress [60]. MiR‐221 plays a role in endothelial cell function and inhibits pro‐apoptotic signalling [61], whereas miR‐210 is a well‐known hypoxia‐inducible miRNA that promotes cell survival under low oxygen conditions by targeting mitochondrial metabolism and pro‐apoptotic genes [62]. Interestingly, recent evidence suggests that cardiac progenitor cells can also release extracellular vesicles containing miR‐210, which may contribute to its elevated levels in the circulation following myocardial infarction [63]. These findings imply that circulating miR‐210 may originate not only from stressed cardiomyocytes but also from resident progenitor cells engaging in paracrine cardioprotection. In contrast, miR‐410‐5p has been reported to inhibit cardiomyocyte autophagy and angiogenesis, potentially impairing tissue recovery after ischemia [64]. MiR‐532 may contribute to pro‐inflammatory responses and metabolic dysregulation in the diabetic heart [65, 66]. MiR‐34a is well‐known for promoting cellular senescence and apoptosis by targeting SIRT1 and Bcl‐2, both of which are critical regulators of cardiac cell survival [67]. These miRNAs may be associated with coordinated regulation of AMPK‐related signalling and maintain cellular homeostasis in ischemic myocardium. However, the integration of circulating miRNA profiles with transcriptomic data was not performed in the present study, and the direct regulatory interactions between individual miRNAs and AMPK signalling were neither validated. Therefore, the potential downstream targets and regulatory networks linking these miRNAs to the observed gene expression changes remain to be elucidated. Future studies incorporating miRNA target prediction, transcriptomic integration, and network‐based analyses will be important to further clarify these relationships and strengthen mechanistic insights.

One limitation of this study is that the exact cellular origin and delivery mechanisms of these circulating miRNAs remain incompletely defined. Although cardiomyocytes, endothelial cells, and other cell types may contribute, direct evidence supporting their specific sources and inter‐organ transfer pathways is lacking. Therefore, the proposed rIPC–circulating miRNA–heart communication axis should be considered hypothetical and requires further experimental validation. Furthermore, the mechanisms by which rIPC and diabetes modulate serum miRNA expression remain to be elucidated. Possible regulatory pathways include changes in miRNA transcription, exosomal packaging, or release due to cellular stress or injury. Our study did not address whether these miRNAs affect other organs beyond the heart. Given their presence in circulation, it is reasonable to hypothesize systemic effects on renal, hepatic, or neural tissues, which warrants further investigation. Future studies should aim to (1) trace the cellular sources of these miRNAs using lineage‐labelling or cell‐specific knockout models; (2) clarify their upstream regulators in the context of rIPC and diabetes; and (3) determine their target genes and signalling pathways in cardiomyocytes and beyond. Importantly, the functional evidence supporting the role of these miRNAs is primarily derived from in vitro experiments. Therefore, the causal contribution of specific circulating miRNAs to rIPC‐mediated cardioprotection in vivo remains uncertain and requires further investigation. Accordingly, the present study should be interpreted as identifying molecular signatures associated with rIPC efficacy rather than establishing direct mechanistic causality. Attempts to combine systemic administration of miRNA mimics with pharmacological AMPK inhibition resulted in severe toxicity and early mortality in our pilot experiments. These findings suggest that such approaches may induce non‐physiological perturbations and limit interpretability in vivo. Therefore, causal validation using this strategy was not pursued further. Moreover, most in vitro experiments are conducted in H9c2 cells, which, although widely used, do not fully recapitulate the phenotype and functional characteristics of mature primary cardiomyocytes. Validation in primary cardiomyocytes or iPSC‐derived cardiomyocytes would strengthen the translational relevance of our findings. Second, all analyses are performed at single‐time‐point endpoints, which may not capture the dynamic changes of circulating miRNAs or AMPK signalling over time. Third, although scramble controls are included for both miRNA mimics and sponges, potential off‐target effects cannot be entirely excluded and other regulatory pathways may also contribute to the observed outcomes. Another limitation of this study is that only male rats were used. Sex differences in cardiovascular physiology and ischemic injury are well recognized, and female animals may exhibit different responses to rIPC, potentially influenced by hormonal regulation. Therefore, future studies including both sexes will be necessary to generalize these findings. Future studies using multiple time points, more physiological cardiomyocyte models and comprehensive target validation approaches would help address these limitations.

In conclusion, our findings provide new evidence suggesting an association between rIPC‐induced cardioprotection and specific circulating miRNAs and suggest a potential opposing association of diabetes‐associated miRNAs in influencing this effect. These findings provide a foundation for future mechanistic studies and may inform the identification of miRNAs as potential biomarkers or modulators of cardioprotection in diabetic conditions.

## Author Contributions

**Bang'e Zhao:** validation, software. **Yeyi Bai:** investigation, methodology, validation. **Haijian Liu:** writing – review and editing, writing – original draft, funding acquisition, project administration. **Tingting Liu:** software, methodology. **Bei Tian:** formal analysis, data curation.

## Funding

This project is supported by Pudong New Area Science and Technology Development Fund (PKJ2024‐Y49) and Pudong New Area Medical Discipline Construction Project (Important Discipline) (PWZxq2022‐11).

## Conflicts of Interest

The authors declare no conflicts of interest.

## Supporting information


**Figure S1:** Validation of lentiviral transduction efficiency in H9c2 cells. Representative images of H9c2 cells transduced with blank pCDH‐CMV‐MCS‐EF1‐puro lentiviral vector. Bright‐field (top), GFP fluorescence (488 nm, middle) and merged images (bottom) are shown. GFP‐positive cells indicate successful transduction. These images are representative of independent experiments. Scale bar, 100 μm.

**Table S1:** Information of reagents used in this study.

**Table S2:** Summary of animal inclusion, exclusion, and survival in each experimental group.

**Table S3:** Differentially expressed miRNAs compared bewteen AMI + rIPC vs. AMI.

**Table S4:** Differentially expressed miRNAs compared bewteen DAMI + rIPC vs. DAMI.

**Table S5:** Differentially expressed genes compared between hypoxic H9c2 cells treated with and without miR‐19a‐3p mimics.

**Table S6:** Differentially expressed genes compared between hypoxic H9c2 cells treated with and without miR‐221‐5p mimics.

**Table S7:** Differentially expressed genes compared between hypoxic H9c2 cells treated with and without miR‐210‐5p mimics.

**Table S8:** Differentially expressed genes compared between hypoxic H9c2 cells treated with and without miR‐410‐5p mimics.

**Table S9:** Differentially expressed genes compared between hypoxic H9c2 cells treated with and without miR‐532‐5p mimics.

**Table S10:** Differentially expressed genes compared between hypoxic H9c2 cells treated with and without miR‐34a‐3p mimics.

## Data Availability

The data that support the findings of this study are available from the corresponding author upon reasonable request.
