# Supplementary figures and images for: Circulating miRNAs Correlate With rIPC‐Induced Cardioprotection and Its Impairment in Diabetic Myocardial Infarction via AMPK Signalling
Source: J Cell Mol Med. 2026 May 13;30(10):e71163. doi: 10.1111/jcmm.71163 (PMC13171722; doi:10.1111/jcmm.71163)

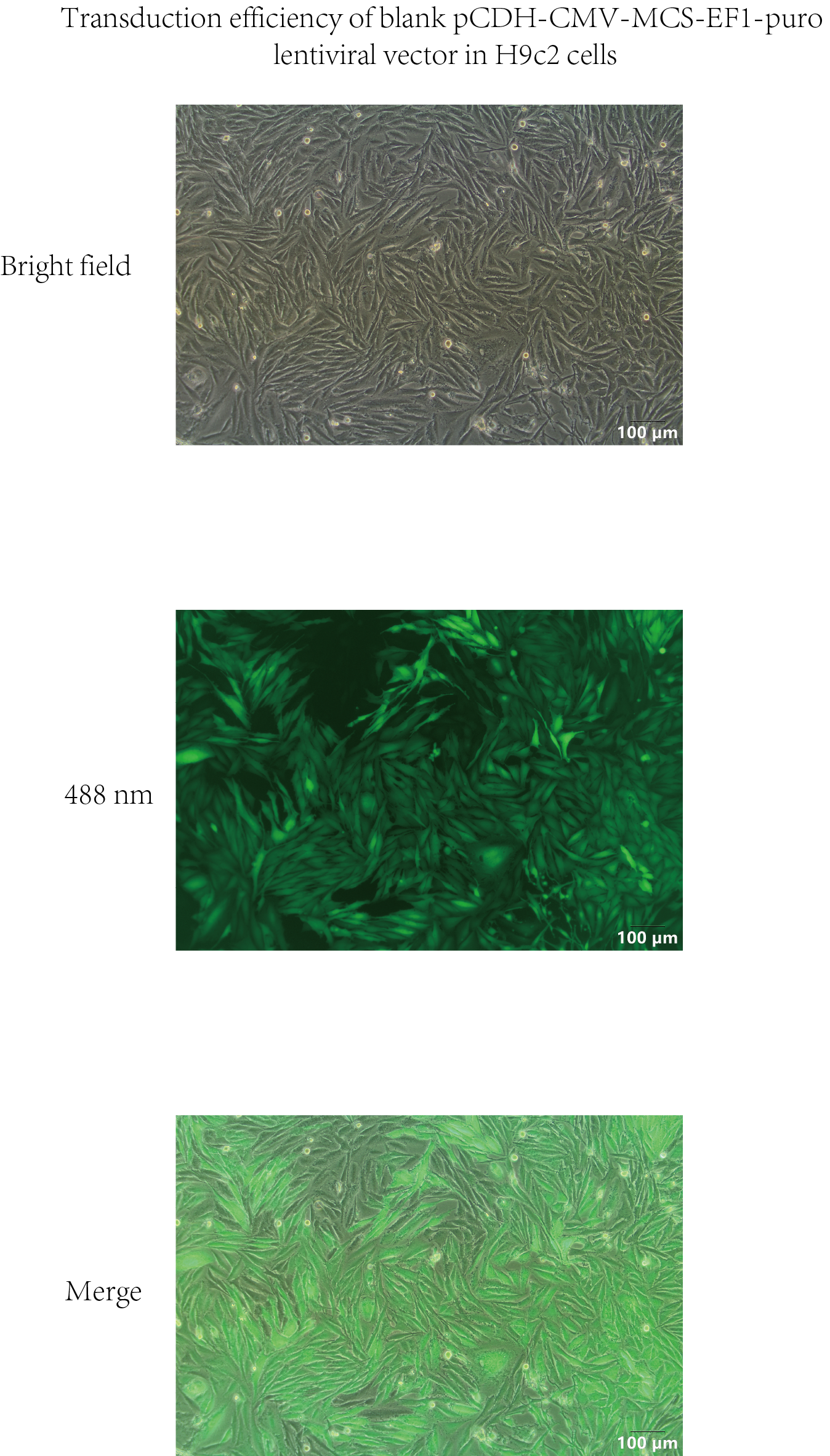

Supplement: Supplementary file 1 — Figure S1: Validation of lentiviral transduction efficiency in H9c2 cells. Representative images of H9c2 cells transduced with blank pCDH‐CMV‐MCS‐EF1‐puro lentiviral vector. Bright‐field (top), GFP fluorescence (488 nm, middle) and merged images (bottom) are shown. GFP‐positive cells indicate successful transduction. These images are representative of independent experiments. Scale bar, 100 μm. [file JCMM-30-e71163-s007.tif]
