# Supplementary material for: Circulating miRNAs Correlate With rIPC‐Induced Cardioprotection and Its Impairment in Diabetic Myocardial Infarction via AMPK Signalling
Source: J Cell Mol Med. 2026 May 13;30(10):e71163. doi: 10.1111/jcmm.71163 (PMC13171722; doi:10.1111/jcmm.71163)
Supplement: Supplementary file 2 — Table S1: Information of reagents used in this study. [file JCMM-30-e71163-s005.docx]

| Reagents | Manufacturer | Country | Catalog Number |
| --- | --- | --- | --- |
| Streptozotocin | APExBIO | Houston, TX, USA | A4457 |
| AICAR | APExBIO | Houston, TX, USA | C4617 |
| TNF-α ELISA Kit | Elabscience | Wuhan, China | E-EL-R2856 |
| IL-6 ELISA Kit | Elabscience | Wuhan, China | E-EL-R0015 |
| IL-1βELISA Kit | Elabscience | Wuhan, China | E-EL-R0012 |
| CK-MB ELISA Kit | Elabscience | Wuhan, China | E-EL-R1327 |
| MDA ELISA Kit | Elabscience | Wuhan, China | E-EL-0060 |
| TTC solution | MedChemExpress | Shanghai, China | HY-D0714 |
| H&E staining Kit | MedChemExpress | Shanghai, China | [HY-K0315](https://www.medchemexpress.cn/inhibitor-kit/hematoxylin-eosin-staining-kit.html) |
| HIF-1α primary antibody | ABclonal | Wuhan, China | A11945 |
| Bax primary antibody | ABclonal | Wuhan, China | A19684 |
| Bcl-2 primary antibody | ABclonal | Wuhan, China | A25812 |
| IL-10 primary antibody | ABclonal | Wuhan, China | A2171 |
| HRP-conjugated goat anti-rabbit IgG (H+L) | ABclonal | Wuhan, China | AS014 |
| AMPKα1 primary antibody | ABclonal | Wuhan, China | A28094 |
| p-AMPKα (Thr172) primary antibody | Cell Signaling Technology | Danvers, MA, USA | 50081 |
| LC3 primary antibody | Proteintech | Wuhan, China | 14600-1-AP |
| mTOR primary antibody | ABclonal | Wuhan, China | A2445 |
| p-mTOR (Ser2448) primary antibody | Proteintech | Wuhan, China | 67778-1-Ig |
| p62 primary antibody | ABclonal | Wuhan, China | A19700 |
| GAPDH primary antibody | ABclonal | Wuhan, China | AC002 |
| Bcl-2 primary antibody | Proteintech | Wuhan, China | 68103-1-Ig |
| Bax primary antibody | Proteintech | Wuhan, China | 50599-2-Ig |
| CoraLite Plus 488-Goat anti-rabbit recombinant secondary antibody | Proteintech | Wuhan, China | RGAR002 |
| CoraLite Plus 594-Goat anti-mouse recombinant secondary antibody | Proteintech | Wuhan, China | RGAM004 |
| DAB peroxidase substrate kit | MedChemExpress | Shanghai, China | HY-K1107 |
| TRIzol | Thermo Fisher Scientific | Waltham, MA, USA | 15596026CN |
| Ion Torrent NGS Reverse Transcription Kit | Thermo Fisher Scientific | Waltham, MA, USA | A45003 |
| Collibr Stranded RNA Library Prep Kit for Illumina Systems with Human/Mouse/Rat rRNA Depletion Kit | Thermo Fisher Scientific | Waltham, MA, USA | A39003024 |
| miRNeasy Serum/Plasma Kit | Qiagen | Hilden, Germany | 217184 |
| Qubit microRNA Assay Kit | Thermo Fisher Scientific | Waltham, MA, USA | Q32881 |
| miScript II RT Kit | Qiagen | Hilden, Germany | 218160 |
| miScript SYBR Green PCR Kit | Qiagen | Hilden, Germany | 218073 |
| QuantStudio 7 Pro Real-time PCR system | Applied Biosystems  Thermo Fisher Scientific | Waltham, MA, USA | A43183 |
| miScript Primer Assay | Qiagen | Hilden, Germany | Customized |
| NEBNext Small RNA Library Prep Set for Illumina | New England Biolabs | Ipswich, MA, USA | E7330 |
| H9c2 cells | ATCC | Manassas, VA, USA | CRL-1446 |
| DMEM | Thermo Fisher Scientific | Waltham, MA, USA | 11965092 |
| FBS | Thermo Fisher Scientific | Waltham, MA, USA | 10099141C |
| Penicillin-streptomycin | Thermo Fisher Scientific | Waltham, MA, USA | 15140122 |
| rno-miR-19a-3p mimic | MedChemExpress | Shanghai, China | HY-R00401 |
| [rno-miR-221-5p mimic](https://www.medchemexpress.cn/hsa-mir-221-5p-mimic.html) | MedChemExpress | Shanghai, China | [HY-R04311](https://www.medchemexpress.cn/rno-mir-221-5p-mimic.html) |
| [rno-miR-210-5p mimic](https://www.medchemexpress.cn/hsa-mir-221-5p-mimic.html) | MedChemExpress | Shanghai, China | [HY-R04300](https://www.medchemexpress.cn/rno-mir-210-5p-mimic.html) |
| [rno-miR-410-5p mimic](https://www.medchemexpress.cn/hsa-mir-221-5p-mimic.html) | MedChemExpress | Shanghai, China | [HY-R00920](https://www.medchemexpress.cn/hsa-mir-410-5p-mimic.html) |
| [rno-miR-532-5p mimic](https://www.medchemexpress.cn/hsa-mir-221-5p-mimic.html) | MedChemExpress | Shanghai, China | [HY-R04510](https://www.medchemexpress.cn/rno-mir-532-5p-mimic.html) |
| [rno-miR-34a-3p mimic](https://www.medchemexpress.cn/hsa-mir-221-5p-mimic.html) | MedChemExpress | Shanghai, China | [HY-R04374](https://www.medchemexpress.cn/rno-mir-34a-3p-mimic.html) |
| [rno-miR-133b-5p mimic](https://www.medchemexpress.cn/hsa-mir-221-5p-mimic.html) | MedChemExpress | Shanghai, China | [HY-R04227](https://www.medchemexpress.cn/rno-mir-133b-5p-mimic.html) |
| [rno-miR-143-3p mimic](https://www.medchemexpress.cn/hsa-mir-221-5p-mimic.html) | MedChemExpress | Shanghai, China | [HY-R04234](https://www.medchemexpress.cn/rno-mir-143-3p-mimic.html) |
| [rno-miR-145-5p mimic](https://www.medchemexpress.cn/hsa-mir-221-5p-mimic.html) | MyBioSource | San Diego, CA, USA | MBS8302658 |
| [rno-miR-29b-3p mimic](https://www.medchemexpress.cn/hsa-mir-221-5p-mimic.html) | MedChemExpress | Shanghai, China | [HY-R04636](https://www.medchemexpress.cn/rno-mir-29b-3p-mimic.html) |
| [rno-miR-149-5p mimic](https://www.medchemexpress.cn/hsa-mir-221-5p-mimic.html) | QIAGEN | Hilden, NRW, Germany | MSY0000159 |
| [rno-miR-292-3p mimic](https://www.medchemexpress.cn/hsa-mir-221-5p-mimic.html) | MedChemExpress | Shanghai, China | [HY-R04321](https://www.medchemexpress.cn/rno-mir-292-3p-mimic.html) |
| [rno-miR-221-5p mimic](https://www.medchemexpress.cn/hsa-mir-221-5p-mimic.html) | MedChemExpress | Shanghai, China | [HY-R04311](https://www.medchemexpress.cn/rno-mir-221-5p-mimic.html) |
| [rno-miR-105 mimic](https://www.medchemexpress.cn/hsa-mir-221-5p-mimic.html) | MedChemExpress | Shanghai, China | [HY-R04209](https://www.medchemexpress.cn/rno-mir-105-mimic.html) |
| [rno-miR-144-3p mimic](https://www.medchemexpress.cn/hsa-mir-221-5p-mimic.html) | QIAGEN | Hilden, NRW, Germany | MSY0000436 |
| [rno-miR-146a-5p mimic](https://www.medchemexpress.cn/hsa-mir-221-5p-mimic.html) | MedChemExpress | Shanghai, China | [HY-R04238](https://www.medchemexpress.cn/rno-mir-146b-5p-mimic.html) |
| [rno-miR-200c-5p mimic](https://www.medchemexpress.cn/hsa-mir-221-5p-mimic.html) | MedChemExpress | Shanghai, China | [HY-R04285](https://www.medchemexpress.cn/rno-mir-200c-5p-mimic.html) |
| [rno-miR-103-3p mimic](https://www.medchemexpress.cn/hsa-mir-221-5p-mimic.html) | MedChemExpress | Shanghai, China | HY-R04208 |
| [rno-miR-107-5p mimic](https://www.medchemexpress.cn/hsa-mir-221-5p-mimic.html) | MedChemExpress | Shanghai, China | [HY-R04210](https://www.medchemexpress.cn/rno-mir-107-5p-mimic.html) |
| [rno-miR-223-3p mimic](https://www.medchemexpress.cn/hsa-mir-221-5p-mimic.html) | MedChemExpress | Shanghai, China | [HY-R04313](https://www.medchemexpress.cn/rno-mir-223-3p-mimic.html) |
| [rno-miR-802-5p mimic](https://www.medchemexpress.cn/hsa-mir-221-5p-mimic.html) | MedChemExpress | Shanghai, China | HY-R04582 |
| Lipofectamine 3000 | Thermo Fisher Scientific | Waltham, MA, USA | L3000015 |
| miRNA sponge | Thermo Fisher Scientific | Waltham, MA, USA | Custom-synthesized |
| pCDH-CMV-MCS-EF1-puro lentiviral vector | System Biosciences | Palo Alto, CA, USA | CD510B-1 |
| Polybrene | MedChemExpress | Shanghai, China | HY-112735 |
| Puromycin | Thermo Fisher Scientific | Waltham, MA, USA | A1113803 |
| Click-iT Plus TUNEL assay kit | Thermo Fisher Scientific | Waltham, MA, USA | C10618 |
| Protease and phosphatase inhibitor cocktail | MedChemExpress | Shanghai, China | HY-K0013 |
| Pierce Dilution-Free Rapid Gold BCA Protein Assay kit | Thermo Fisher Scientific | Waltham, MA, USA | A55860 |
| PVDF membrane | Thermo Fisher Scientific | Waltham, MA, USA | 88518 |
| Ultra High Sensitivity ECL Kit | MedChemExpress | Shanghai, China | HY-K1005 |
