# Supplementary material for: Circulating miRNAs Correlate With rIPC‐Induced Cardioprotection and Its Impairment in Diabetic Myocardial Infarction via AMPK Signalling
Source: J Cell Mol Med. 2026 May 13;30(10):e71163. doi: 10.1111/jcmm.71163 (PMC13171722; doi:10.1111/jcmm.71163)
Supplement: Supplementary file 3 — Table S2: Summary of animal inclusion, exclusion, and survival in each experimental group. [file JCMM-30-e71163-s004.docx]

| Group | Initially included | Excluded or replaced | Surviving to endpoint |
| --- | --- | --- | --- |
| NC | 10 | 0 | 10 |
| MI | 12 | 2 | 10 |
| MI+rIPC | 12 | 2 | 10 |
| DMI | 12 | 2 | 10 |
| DMI+rIPC | 11 | 1 | 10 |
| DMI+rIPC+AMPKA | 12 | 2 | 10 |

Table S2. Summary of animal inclusion, exclusion, and survival in each experimental group.
